# Levels of Coenzyme Q_10_ and Several COQ Proteins in Human Astrocytoma Tissues Are Inversely Correlated with Malignancy

**DOI:** 10.3390/biom12020336

**Published:** 2022-02-20

**Authors:** Hsiu-Chuan Yen, Bing-Shian Chen, Si-Ling Yang, Shin-Yu Wu, Chun-Wei Chang, Kuo-Chen Wei, Jee-Ching Hsu, Yung-Hsing Hsu, Tzung-Hai Yen, Chih-Lung Lin

**Affiliations:** 1 Department of Medical Biotechnology and Laboratory Science, College of Medicine, Chang Gung University, Taoyuan 33302, Taiwan; jeffery0158@gmail.com (B.-S.C.); young841113@gmail.com (S.-L.Y.); shinyuwu910068@yahoo.com.tw (S.-Y.W.); gene5517228tv@gmail.com (C.-W.C.); 2 Department of Nephrology, Chang Gung Memorial Hospital at Linkou, Taoyuan 333423, Taiwan; m19570@cgmh.org.tw; 3 Department of Neurosurgery, Chang Gung Memorial Hospital at Linkou, Taoyuan 333423, Taiwan; kuochenwei@cgmh.org.tw; 4 Department of Neurosurgery, New Taipei Municipal Tu Cheng Hospital, Chang Gung Medical Foundation, New Taipei City 236017, Taiwan; 5 School of Medicine, College of Medicine, Chang Gung University, Taoyuan 33302, Taiwan; 6 Department of Anesthesiology, Lotung Poh-Ai Hospital, Yilan 26546, Taiwan; hsujcs@yahoo.com.tw; 7 Department of Neurosurgery, Asia University Hospital, Taichuang 41354, Taiwan; yhhsu11@hotmail.com; 8 Department of Occupational Therapy, Asia University, Taichuang 41354, Taiwan

**Keywords:** astrocytoma, malignancy, coenzyme Q_10_, PDSS2, COQ proteins, citrate synthase, COX II

## Abstract

In a previous study, we reported the alterations of primary antioxidant enzymes and decreased citrate synthase (CS) activities in different grades of human astrocytoma tissues. Here, we further investigated coenzyme Q_10_ (CoQ_10_) levels and protein levels of polyprenyl diphosphate synthase subunit (PDSS2) and several COQ proteins required for CoQ_10_ biosynthesis in these tissues. We found that the level of endogenous CoQ_10_, but not of exogenous α-tocopherol, was higher in nontumor controls than in all grades of astrocytoma tissues. The levels of COQ3, COQ5, COQ6, COQ7, COQ8A, and COQ9, but not of COQ4, were lower in Grade IV astrocytoma tissues than in controls or low-grade (Grades I and II) astrocytomas, but PDSS2 levels were higher in astrocytoma tissues than in controls. Correlation analysis revealed that the levels of CoQ_10_ and COQ proteins were negatively correlated with malignancy degree and positively correlated with CS activity, whereas PDSS2 level was positively correlated with malignancy. Moreover, lower level of mitochondrial DNA-encoded cytochrome *c* oxidase subunit 2 was not only associated with a higher malignancy degree but also with lower level of all COQ proteins detected. The results revealed that mitochondrial abnormalities are associated with impaired CoQ_10_ maintenance in human astrocytoma progression.

## 1. Introduction

Coenzyme Q_10_ (CoQ_10_) is an essential electron carrier in the mitochondrial electron transport chain and the only endogenously synthesized lipid-soluble antioxidant in humans [1,2,3]. Coq1–9 and the less-characterized Coq11 proteins are essential for the terminal biosynthetic pathway of CoQ_6_ in the budding yeast *S. cerevisae*, and many of these Coq proteins form high-molecular-weight protein complexes in mitochondria for efficient CoQ biosynthesis. Coq4 and Coq9 do not have known enzymatic functions, and Coq8 could be an atypical kinase, whereas Coq1, Coq2, Coq3, Coq5, Coq6, and Coq7 have known enzymatic functions in CoQ structure formation [4]. The human orthologs of yeast Coq2–9 proteins are COQ2–9 proteins, whereas the human orthologs of yeast Coq1 protein are two polyprenyl diphosphate synthase subunits (PDSS1 and PDSS2) [4]. In addition, two *COQ8* paralog genes (*COQ8A* and *COQ8B*) exist in humans [1,2]. The restoration of CoQ levels through overexpression of human *PDSS* and *COQ* genes in yeast mutants lacking corresponding genes have been demonstrated [4,5], but the identity and function of the human homolog of yeast Coq11 are uncertain, although a putative protein has been indicated based on sequence analyses [4]. As indicated by several review papers [2,4,6], mutations of *PDSS1*, *PDSS2*, and *COQ2–9* genes (except *COQ3*) have been found to be associated with the disease of primary CoQ_10_ deficiency in humans, which is characterized by heterogeneous clinical phenotypes, including manifestation in the central nervous system. CoQ_10_ treatment can improve the manifestations in those patients. In addition, secondary CoQ_10_ deficiency can be caused by mutations of mitochondrial DNA (mtDNA) or nuclear genes not related to CoQ_10_ biosynthesis.

PDSS and COQ proteins in human cells are poorly characterized. We previously identified specific antibodies with concurrent identification of the exact protein forms for PDSS2, COQ3, COQ4, COQ5, COQ6, COQ7, COQ8A, and COQ9 proteins through the gene knockdown approach in the human 143B cell line [7,8,9]. Three PDSS2 isoforms (PDSS2-a, PDSS2-b, and PDSS-c) and two COQ3 isoforms (COQ3-a and COQ3-b) were identified though Western blotting. On the basis of the approximate sizes of the observed bands on Western blotting and predicted molecular masses, we proposed that these proteins, except COQ9 and PDSS2-c, are mature forms generated through the removal of putative mitochondrial targeting sequences (MTSs) from predicted full-length precursor proteins [7]. Search results on the Uniprot webpage for human PDSS2 (Entry ID: Q86YH6) indicate that alternative splicing can produce two major protein isoforms, with molecular masses of 44 and 26 kDa, which should correspond to the precursor form of PDSS2-b and the PDSS2-c band detected, respectively. We speculated that PDSS2-a and COQ3-a could be isoforms resulting from posttranslational modifications of PDSS2b and COQ3-b, respectively, although this was not verified. In addition, we showed that the proteins detected were mitochondrial proteins, and many of them were present in high-molecular-weight protein complexes in the mitochondria, although we could not determine whether they were associated with the same complex [7,9]. Furthermore, on the basis of our previous findings, we hypothesized that oxidative stress increases CoQ_10_ levels in human cells because of upregulation of PDSS and COQ genes, whereas severe mitochondrial energy deficiency, such as during treatment with a mitochondrial uncoupler or in cybrids with pathological mutations of mtDNA, impairs the import of PDSS and COQ precursor proteins into mitochondria, the formation of mature proteins without MTSs in the mitochondria, or the assembly of putative PDSS or COQ protein complexes in the mitochondria of human cells. That could be the underlying mechanism for secondary CoQ_10_ deficiency under conditions with mitochondrial dysfunction [7,8,9,10,11].

Astrocytoma, a type of glioma, is a tumor derived from astrocytes and is commonly found in human brain tumors. It is classified as Grade I–IV depending on its malignancy. The complete removal of Grade II–IV astrocytoma through surgery is difficult, and patients with Grade IV astrocytoma usually only have a median survival time of less than 1 year [12,13,14]. Mitochondrial dysfunction has been indicated to play a major role in the formation and progression of cancers [15]. Few studies have indicated mitochondrial abnormalities in human astrocytomas, such as ultrastructural abnormalities [16,17] and decreased activities of mitochondrial respiratory complexes in high-grade astrocytomas [18]. Furthermore, decreased copy number of mtDNA [18] and mtDNA mutations [19] were found in Grade IV astrocytomas. We previously reported the alterations of mRNA levels, protein levels, and activities of various primary antioxidant enzymes in different grades of astrocytomas versus nontumor controls from patients [20]. Moreover, a decrease in the activity of citrate synthase (CS), an enzyme of the tricarboxylic acid cycle in the mitochondrial matrix, was found to be associated with a high degree of malignancy in the same study [20], a result similar to the findings of Feichtinger et al. [19].

To the best of our knowledge, although a few studies have determined CoQ_10_ levels or investigated *PDSS* genes, PDSS proteins, and *COQ* genes in some human cancer tissues, no studies have examined brain tumors or detected COQ proteins in them. So far, CoQ_10_ levels were found to be lower only in the tissues of breast cancer tissues [21] and hepatocellular carcinoma [22] compared with the surrounding normal tissues. Several studies have examined *PDSS* genes or PDSS proteins. Immunohistochemistry (IHC) results have revealed low PDSS2 protein levels in the tissues of hepatocellular carcinoma [22,23], gastric cancer [24], and melanoma [25]. *PDSS2* expression was significantly decreased in the tissues of gastric cancer [24] and lung cancers [26] compared with the adjacent normal tissues. A recent study reported upregulated *PDSS1* expression and PDSS1 protein levels in triple-negative breast cancer tissues [27]. Regarding *COQ* genes, only one study has reported the association of high *COQ3* expression with poor prognosis of esophageal squamous cell carcinoma [28].

In the present study, we hypothesized that CoQ_10_ levels and protein levels of PDSS and COQ proteins would decrease in astrocytomas because of mitochondrial dysfunction. Therefore, we investigated levels of endogenous CoQ_10_ versus exogenous α-tocopherol (vitamin E) and of PDSS2 and several COQ proteins in the same control and astrocytoma tissues as used in the previous study [20]. Furthermore, the associations of various analytical results with the malignancy degree and 1-year outcome, based on Glasgow Outcome Scale (GOS) scores, were evaluated through a correlation analysis. In addition, the correlations of various parameters with CS activity and level of mtDNA-encoded cytochrome *c* oxidase subunit 2 (COX II) were analyzed to indirectly examine the associations between possible mitochondrial abnormalities and the status of endogenous CoQ_10_ and its biosynthetic proteins.

## 2. Materials and Methods

### 2.1. Background Information of Patients and Astrocytoma Specimens

In this study, we used tissues samples from the 40 patients included in the previous study investigating antioxidant enzymes [20]. All the patients were treated at the Department of Neurosurgery of Chang Gung Memorial Hospital at Linkou, and brain tissues were removed from the patients during the necessary surgery. Among the 40 patients, 5 patients had traumatic brain injury (nontumor controls), 9 patients had low-grade astrocytomas (2 and 7 patients with Grade I and Grade II tumor, respectively), 6 patients had Grade III astrocytomas, and 20 patients had Grade IV astrocytomas. In this study, the CoQ_10_ and α-tocopherol levels of all the patients were measured, but two samples from patients with Grade III astrocytomas could not be analyzed for PDSS2 and COQ proteins because the amount of samples was not sufficient. All astrocytoma tissues used were primary tumor tissues that had not been subjected to radiotherapy or chemotherapy. The histological criteria for astrocytoma classification were based on the World Health Organization guidelines of 2007 [13] because the tissues were collected from 2005 to 2007. Because the R132H mutation of isocitrate dehydrogenase 1 is a new marker for glioma classification [29], this mutation was identified through Western blotting in our previous study [20]. GOS in five grades was used to evaluate the outcomes of patients 1 year after surgery: good recovery and resumption of normal life (G); independent lifestyle with moderate disability (M); severe disability and dependent for daily support (S); persistent vegetative state (V); and death (D). Table 1 summarizes the patient information and the analyses performed in this study for each coded patient that was included. Furthermore, samples that were previously analyzed for CS activity [20] are also indicated in the Table 1 because the data were included in this study for comparison or statistical analyses.

### 2.2. Simultaneous Detection of CoQ_10_ and α-Tocopherol Levels in Astrocytoma Tissues through High-Performance Liquid Chromatography

In a previous study, we established the methods for the simultaneous analysis of CoQ_10_ and multiple lipid-soluble antioxidants—including α-tocopherol, a major vitamer of vitamin E in human plasma; this method involves using unique HPLC with the coulometric array detector (CoulArray HPLC) from ESA Biosciences, Inc., Chelmsford, MA, USA [30]. In this study, we adopted these procedures to detect CoQ_10_ and α-tocopherol levels in human brain tissues. Tissues were first homogenized in 50 mM sodium phosphate buffer (pH 7.4) in a glass homogenizer with frosted surface. After mixing 0.1 mL of the homogenate with 10 μL of the internal standard retinyl acetate (RA), the homogenate was mixed with an equal amount of 2.5% sodium dodecyl sulfate (SDS), according to the method of Leray et al. [31]. Subsequently, 0.2 mL of the SDS-containing homogenate was mixed with 0.2 mL of ethanol containing butylated hydroxyanisole (BHA); this was followed by extraction with 1 mL of hexane through vortexing for 10 min and centrifuging at 4000× *g* for 10 min. The upper layer with hexane was transferred to a new microcentrifuge tube. The hexane extraction step was repeated. The combined hexane layers were evaporated to dryness under nitrogen gas, dissolved in ethanol containing BHA, filtered through a syringe filter, and then subjected to HPLC analysis.

**Figure 1:**
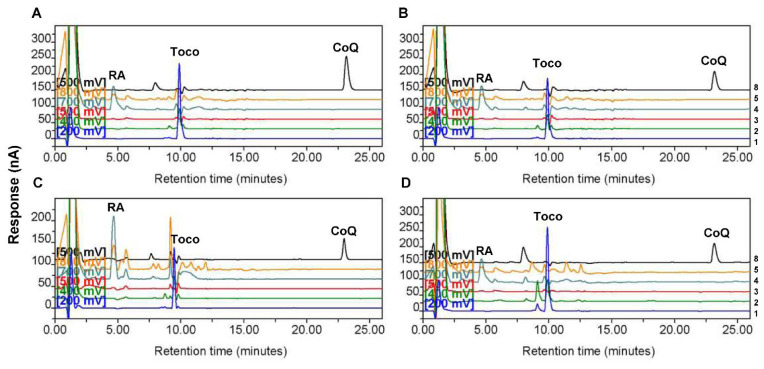
Representative high-performance liquid chromatography chromatograms showing the peaks of retinyl acetate (RA), α-tocopherol, and coenzyme Q_10_ (CoQ_10_) in control and astrocytoma tissues. Chromatograms for one tissue sample from (**A**) non-tumor control, (**B**) low-grade astrocytoma, (**C**) Grade III astrocytoma, and (**D**) Grade IV astrocytoma groups. The numbers at the right side indicate the channel numbers. The chromatograms for Channels 1 (200 mV), 2 (400 mV), 3 (500 mV), 4 (700 mV), 5 (800 mV), and 8 (500 mV) are indicated by the color of blue, light green, red, dark green, yellow, and black, respectively. The chromatograms for Channels 6 and 7 (−1000 mV) are not shown. RA indicates the dominant peak of RA, the internal standard, on Channel 4 (700 mV). Toco and CoQ indicate the dominant peaks of α-tocopherol and CoQ_10_ on Channel 1 (200 mV) and Channel 8 (500 mV), respectively.

The same electrical potentials for the eight channels on the detector (200, 400, 500, 700, 800, −1000, −1000, and 500 mV), gradient elution methods, and analytical methods described previously for human plasma [30] were then applied for the detection for tissues through HPLC in the present study. The MD-150 C18 column from ESA and the separation based on reverse-phase chromatography were used. The compositions of the mobile phases were also as described previously [30]. Total CoQ_10_ levels, which included both oxidized and reduced forms of CoQ_10_, were detected because the reduced form was easily oxidized during the extraction procedure and no peak corresponding to the reduced form could be detected. Representative chromatograms from the four groups—illustrating simultaneous detection of RA, α-tocopherol, and CoQ_10_—are presented in Figure 1. Channel 4 (700 mV) and Channel 2 (400 mV) were the dominant channels exhibiting the major peaks of RA and α-tocopherol, respectively, due to their oxidation on electrodes. CoQ_10_ in oxidized form in the samples was first reduced on Channels 6 and 7 (−1000 mV) and then oxidized again on Channel 8 (500 mV). The height ratio of the α-tocopherol or CoQ_10_ peak to the RA peak was used either for establishing the calibration curve or for quantification of unknown samples by using the CoulArray for Windows 32 software (Version 3.1) [32]. In this way, the difference in the recovery after sample extraction could be normalized. The protein concentrations of the remaining homogenates were determined using the Bio-Rad Protein Assay kit (Bio-Rad, Hercules, CA, USA). The values of CoQ_10_ and α-tocopherol levels were normalized by the amount of protein for each sample.

### 2.3. Detection of PDSS2 and Various COQ Proteins in Astrocytoma Tissues through Western Blot Analysis

We used the same tissue homogenates used in the previous study for detecting the protein levels of antioxidant enzymes [20]. Furthermore, the Western blot procedure described in that study [20] was applied in the present study. In brief, proteins were separated using SDS-polyacrylamide gel electrophoresis and were then transferred to nitrocellulose membranes (PerkinElmer, Waltham, MA, USA). The PageRuler Prestained Protein Ladder from Thermo Scientific (#26616) and Precision Plus Protein Dual Color Standards from Bio-Rad (#161-0374) were loaded in each blot as protein molecular weight markers. The nitrocellulose membranes were reacted with primary antibodies at 4 °C overnight, reacted with horseradish peroxidase (HRP)-labeled secondary antibodies at room temperature for a few hours, and then incubated with Immobilon Western Chemiluminescent HRP substrate (Millipore, Burlington, MA, USA) or SuperSignal West Femto Maximum Sensitivity Substrate (Thermo Fisher, Waltham, MA, USA). Chemiluminescent signals were recorded using the Amersham Imager 600 system (GE Healthcare, Chicago, IL, USA) and signal intensity was quantified using Multi Gauge software version 3.0 (Fujifilm, Tokyo, Japan) [33]. Actin was detected as the loading control. The samples were divided into four separate Western blotting experiments for the same protein. One control sample (#2) was used as the reference sample to be loaded for different blots. The intensity of target proteins was normalized by the intensity of actin, and the data are presented in relation to the reference sample in each blot. The commercial sources of antibodies for detecting human PDSS2, COQ3, COQ4, COQ6, COQ7, COQ8A/CABC1, COQ9, COX II, and actin were indicated in our previous publication [7]. The COQ5 antibody was previously generated in our laboratory [8]. PDSS1, COQ2, and COQ8B were not detected because we have not found reliable commercial antibodies that can be verified using the gene knockdown approach. The PDSS2-a band was not quantified because it could not be easily separated from the PDSS2-b band and the corresponding abundance was low [7].

### 2.4. Data Presentation and Statistical Analysis

Data were divided into four groups for the comparison. These four groups are nontumor control brain tissues, low-grade (both Grade I and Grade II) astrocytomas, Grade III astrocytomas, and Grade IV astrocytomas. For various parameters, the data are presented as box plots created using SigmaPlot version 10.0.1 (Systat Software Inc., San Jose, CA, USA) [34]. Statistical analysis was performed using IBM SPSS Statistics version 22.0 software (IBM, Armonk, NY, USA) [35]. The nonparametric *Mann–Whiney U* test was used to determine the statistical significance for the differences between any two groups for all the measured parameters. The nonparametric Spearman’s correlation analysis was conducted to examine the significance of the correlations between the malignancy degree (1 to 4 for the groups of nontumor controls, low grades, Grade III, and Grade IV, respectively) or GOS grade (“G” = 1, “M” = 2, “S” = 3, “V” = 4, and “D” = 5) and various parameters. Differences were considered statistically significant when *p* values were less than 0.05.

## 3. Results

### 3.1. Levels of CoQ_10_, but Not α-Tocopherol, Were Higher in the Nontumor Control Group Than in All Astrocytoma Groups

**Figure 2:**
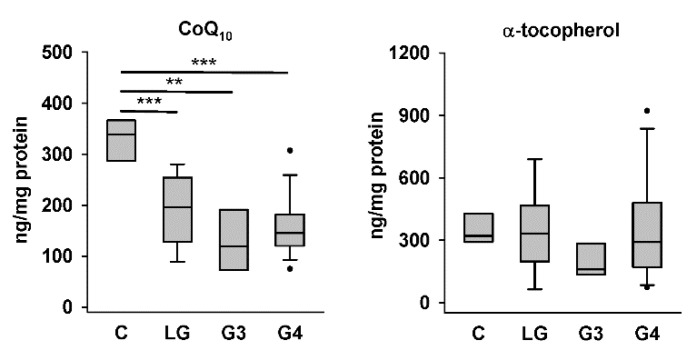
CoQ_10_ and α-tocopherol levels in control and astrocytoma tissues. C, controls; LG, low-grade astrocytomas; G3, Grade III astrocytomas; and G4, Grade IV astrocytomas. ** *p* < 0.01; *** *p* < 0.005.

We first compared CoQ_10_ and α-tocopherol levels in the four groups of tissue homogenates. The HPLC results showed that the levels of endogenously synthesized CoQ_10_ in low-grade, Grade III, and Grade IV astrocytoma tissues were significantly lower than those in nontumor control tissues, whereas the levels of exogenously acquired α-tocopherol in the same samples were not different among the four groups (Figure 2). Representative chromatograms for one sample each from the control group and different astrocytoma groups are shown in Figure 1. The higher abundance of CoQ_10_ relative to that of α-tocopherol in the sample from the control group (Figure 1A) than that in samples from astrocytomas, particularly that from the group of Grade IV astrocytomas (Figure 1D), could be observed.

### 3.2. Levels of PDSS2, Various COQ Proteins, and COX II in Different Groups

**Figure 3:**
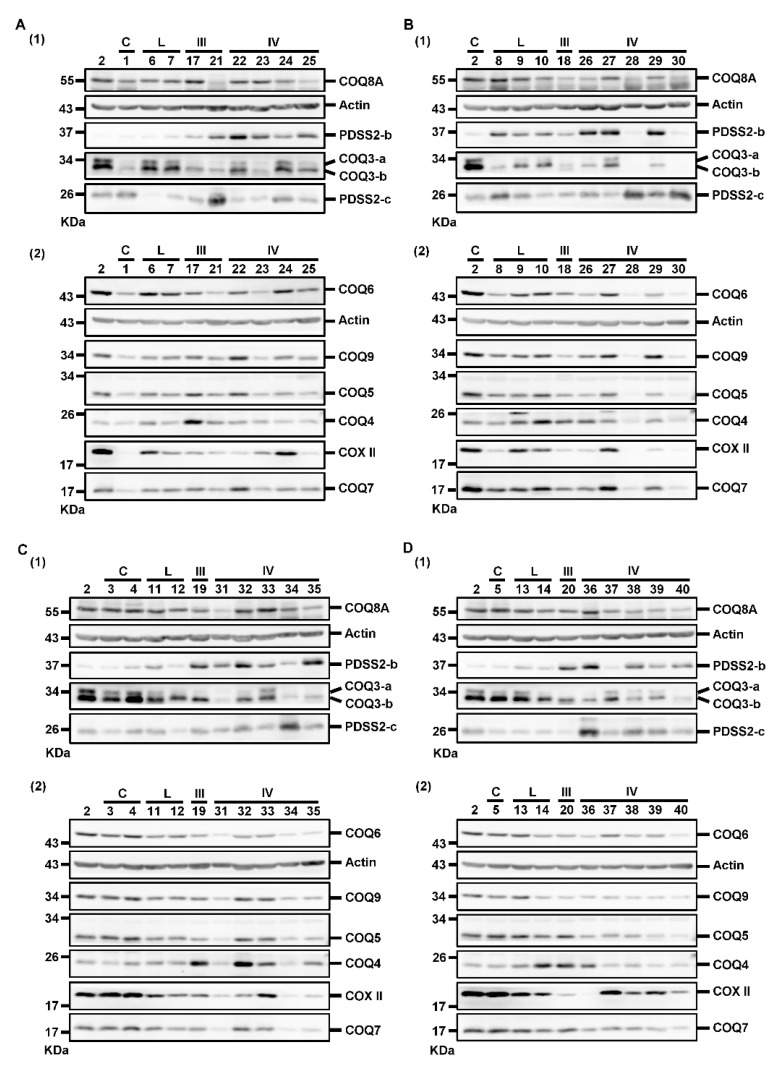
Western blotting results for detecting the levels of PDSS2 and various COQ proteins. Samples were divided into four groups (**A**–**D**) for Western blotting. For each group, samples were analyzed in two blots, blot (1) and blot (2), to detect all the target proteins. The #2 sample was used as the reference sample for comparison across different blots. The numbers above each blot indicate the patient number. The numbers at the left side of each blot indicate the molecular mass of the marker bands of the protein sizing markers from Thermo or Bio-Rad. kDa, kilodaltons; C, controls; L, low-grade astrocytomas; III, Grade III astrocytomas; IV, Grade IV astrocytomas.

We further investigated whether the decreased CoQ_10_ levels in astrocytomas was related to the alterations of PDSS2 and COQ proteins. Moreover, COX II level was detected as an additional indicator of mitochondrial status. Actin was detected as a loading control for the normalization of target protein abundance during quantification. The results of Western blotting divided into eight blots are illustrated in Figure 3. Samples were divided into four sets of Western blotting experiments for the same protein. The homogenate of sample #2 sample was analyzed as the reference sample for normalization of the quantification results across the four sets of results.

**Figure 4:**
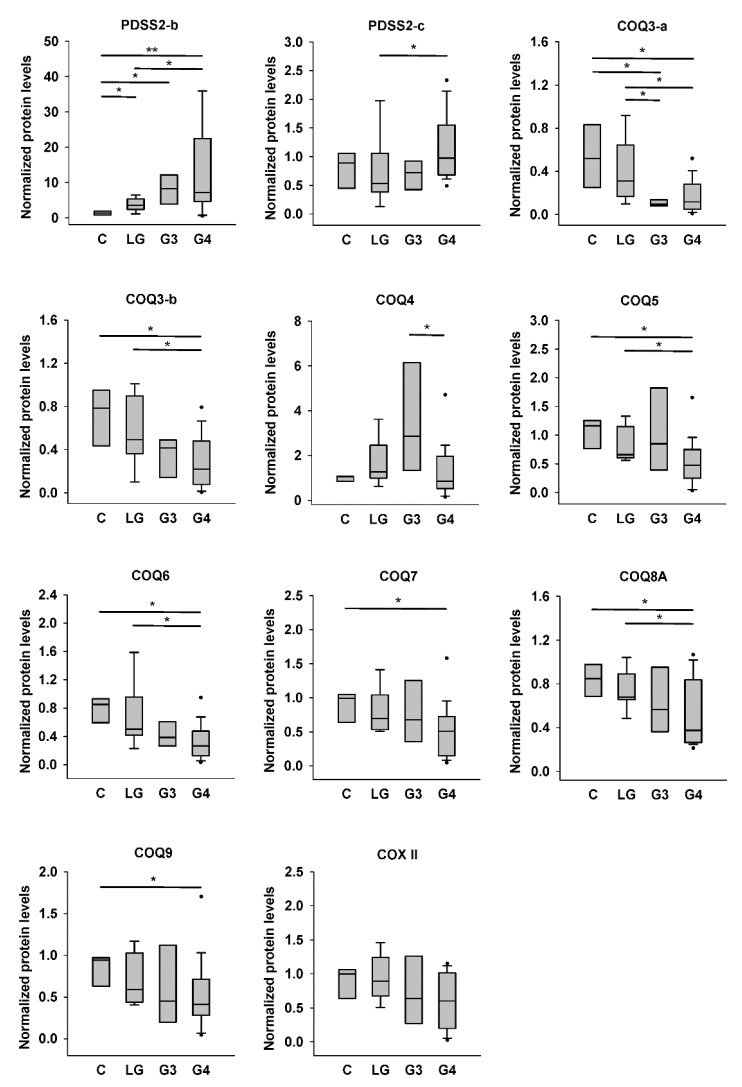
Quantification results for PDSS2 and COQ protein levels in control and astrocytoma tissues. Protein densities shown in Figure 3 were quantified. Intensities of target proteins of each sample were normalized by the intensity of actin in the same blot. The actin-normalized protein levels of each sample were then further normalized by that of the same reference sample (sample #2) in each blot. C, controls; LG, low-grade astrocytomas; G3, Grade III astrocytomas; G4, Grade IV astrocytomas. * *p* < 0.05; ** *p* < 0.01.

The quantification results are shown in Figure 4. We found significantly higher PDSS2-b level in low-grade, Grade III, and Grade IV astrocytomas than in nontumor controls, and those in Grade IV astrocytomas were also higher than those in low-grade tumors. However, PDSS2-c level was significantly higher only in Grade IV astrocytomas compared with that in low-grade tumors. COQ3-a levels in Grade III and Grade IV astrocytomas were lower than those in low-grade tumors and nontumor controls. COQ3-b level showed a similar tendency, but only that in Grade IV astrocytomas was significantly lower than those in nontumor controls and low-grade tumors. There was a gradual increase of COQ4 level from control to Grade III astrocytomas, although there was no difference among these three groups. However, the COQ4 level in Grade IV astrocytomas was significantly lower than that in Grade III tumors but similar to that in controls or low-grade tumors. COQ5, COQ6, and COQ8A levels in Grade IV astrocytomas were significantly lower than those in low-grade tumors and nontumor controls. COQ7 and COQ9 levels in Grade IV astrocytomas were significantly lower than those in nontumor controls. Finally, the COX II level did not differ among the four groups.

### 3.3. Correlations of CS Activity or COX II Level with Levels of Various Molecules Detected in This Study

Because CS activity and mtDNA-encoded COX II level might represent different aspects of mitochondrial status, we further examined whether the CS activity previously measured [20] and COX II level had any correlations with the levels of CoQ_10_, PDSS2 protein, or various COQ proteins for all samples. The results presented in Table 2 indicate that CoQ_10_ level was positively correlated with CS activity, but not COX II levels. Moreover, the protein levels of COQ3-a, COQ3-b, COQ5, COQ6, COQ7, COQ8A, and COQ9 were positively correlated with either CS activity or COX II level, whereas PDSS2-c level was inversely correlated with either CS activity or COX II level. There was no correlation between PDSS2-b level and CS activity or COX II level. A positive correlation was noted only between COX II level and COQ4 protein level. As expected, α-tocopherol level was uncorrelated with CS activity or COX II level. However, there was also a positive correlation between CS activity and COX II level.

### 3.4. Correlations of Various Analytes with Malignancy and with GOS Scores for Grade IV Astrcocytomas

The results of the correlation analysis for the correlations of various analytes with malignancy in four levels (non-tumor controls, low-grade astrocytomas, Grade III astrocytomas, and Grade IV astrocytomas) are shown in Table 3. The results showed that CoQ_10_ level, but not α-tocopherol level, was significantly inversely correlated with high malignancy grades. Moreover, the levels of COQ3-a, COQ3-b, COQ5, COQ6, COQ7, COQ8A, COQ9, and COX II levels were negatively correlated with malignancy. However, PDSS2-b and PDSS2-c levels were positively correlated with high malignancy grades. In addition, because CoQ_10_ levels in Grade IV astrocytoma appeared to be slightly higher than those in Grade III astrocytomas, we also performed the same correlation analysis for the data when the Grade IV astrocytoma group was excluded. The results indicate that there was a stronger inverse correlation between CoQ_10_ level and malignancy when only the groups of controls, low-grade astrocytoma, and Grade III astrocytoma were included. The correlation of PDSS2-b or COQ3-a level with malignancy remained significant when excluding Grade IV astrocytomas, but the correlation became nonsignificant for all other parameters. Furthermore, we previously investigated the correlations of various parameters with 1-year GOS score only for the Grade IV astrocytoma group when investigating antioxidant enzymes because high tumor grades were strongly associated with worse 1-year GOS, as expected [20]. Therefore, we further examined whether a correlation existed between various analytes detected in this study with GOS scores only for the Grade IV astrocytoma group. However, we did not discover any significant correlation (Table 3).

Because alterations of CoQ_10_ levels, PDSS2 protein levels, and COQ protein levels in astrocytomas might be associated with mitochondrial abnormalities related to the changes in CS activity or COX II level, we further compared the changes of these molecules after the values were normalized by CS activity or COX II level, which are shown in Figures S1 and S2, respectively, Supplementary Materials. After the normalization by CS activity, the tendency of higher PDSS2-b level in high-grade astrocytomas versus controls or low-grade astrocytomas, higher PDSS2-c level in Grade IV astrocytomas, or lower COQ3-a level in Grade III astrocytomas versus low-grade astrocytomas remains significant. However, other significant changes in CoQ_10_ levels (Figure 2) and other COQ proteins (Figure 4) became nonsignificant (Figure S1, Supplementary Materials). Furthermore, after the normalization by COX II level, similar tendency for normalized PDSS2-b, PDSS2-c, COQ3-a, COQ3-b, COQ4, COQ6 levels (Figure S2, Supplementary Materials) as that for unnormalized levels (Figure 4) could be observed, but that became nonsignificant or less significant. The difference between controls and low-grade astrocytomas for COX II-normalized CoQ_10_ level remains significant. The increase of COQ4 level in Grade III astrocytomas compared with that in controls became significant after the level was normalized by either CS activity or COX II level. Finally, we examined the correlation of the normalized data with malignancy or GOS. The results are shown in Table 4. The conclusion was the same for PDSS2-b, PDSS2-c, COQ3-a, and COQ3-b when normalized by either CS activity or COX II level compared with the results for unnormalized data shown in Table 3. The inverse correlation between malignancy and COQ3-b, COQ5, or COQ6 remains significantly when the data were normalized by COX II. However, CoQ_10_:CS or CoQ_10_:COX II ratio had no correlation with malignancy. It is important to note that COQ4 level became positively correlated with malignancy when the Grade IV astrocytoma group was not included. Moreover, there was an inverse correlation between COQ8A:CS or COQ8A:COX II ratio and 1-year GOS grades for the Grade IV astrocytoma group. Therefore, lower values of COQ8A:CS or COQ8A:COX II ratio, but not other parameters, correlated with worse 1-year outcomes for patients with Grade IV astrocytomas.

## 4. Discussion

This is the first study to demonstrate decreased CoQ_10_ levels and alterations of PDSS and COQ proteins in human astrocytomas or brain tumors. Furthermore, we are the first to investigate alterations of several COQ proteins in human cancer tissues. The alterations were associated with the possible development of mitochondrial abnormalities during progression to a higher astrocytoma grade, which was in line with our hypothesis, although the mechanism for PDSS2 upregulation in astrocytomas remains to be investigated. However, whether PDSS1 and COQ2 proteins that could not be examined in this study play any role in this change is unknown. Unlike many studies investigating CoQ_10_ levels and *PDSS* genes, PDSS proteins, or *COQ* genes in cancers by comparing the results from cancer tissues with those from the surrounding normal tissues [21,22,23,24,26,27], we examined nontumor control brain tissues and astrocytoma tissues of various grades because surrounding tumor tissues might still harbor tumor cells, and the changes could be different among different grades of cancer tissues, as demonstrated by our previous findings on the alterations of antioxidant enzymes in various astrocytoma grades [20].

In our previous study, CS activity was found to be lower in Grade IV astrocytomas than in controls or Grade II astrocytomas, and this lower activity was strongly associated with an increased degree of malignancy [20]. However, unlike changes in CS activity [20], CoQ_10_ level detected in this study was decreased in low-grade astrocytomas and it had a strong inverse correlation with malignancy when the data of Grade IV astrocytomas were not included, suggesting that impaired maintenance of CoQ_10_ level, but not CS or COX II levels, could be associated with the early phase of tumorigenesis. Although CoQ_10_ levels were correlated only with CS activities, the levels of most COQ proteins positively correlated with both CS activities and COX II levels, whereas PDSS2 levels had no correlation with CS activities and COX II levels. In addition, there was a significant correlation between CS activity and COX II level. Similar but different conclusions from data normalized by CS activity or COX II level indicate that multiple factors in the aspect of mitochondrial abnormalities could be related to the changes in CoQ_10_ levels. Because human CS is a mitochondrial matrix enzyme with a MTS [36], its activity may be suppressed by decreased mitochondrial energy production from oxidative phosphorylation because the energy from mitochondrial membrane potential and mitochondrial ATP is in generally required for the import of nuclear DNA-encoded mitochondrial precursor proteins into matrix to form mature proteins after the cleavage of MTSs [37]. This is the same principle we proposed for PDSS and COQ proteins previously [7]. Although COX II level was not found to vary among different groups of tissues, the level was inversely correlated with malignancy, suggesting the presence of mtDNA-related mitochondrial abnormalities. Lower COX II level may cause decreased Complex IV activity and therefore decreased mitochondrial energy production. The findings of concurrent oxidative phosphorylation dysfunction, decreased CS activities, and CoQ deficiency in five different knockout mice with defects in replication, maintenance, transcription, translation, or posttranslational regulation of mtDNA reported by Kuhl et al. [38] further supports this notion. The study of Yubero et al. also indicate that although CoQ_10_ level was correlated with CS activity, CoQ_10_ level was a better biomarker than CS for the defects in enzyme activities of mitochondrial respiratory chain in muscle biopsies from patients with suspected mitochondrial disorders [39]. Nevertheless, possible alterations in mitochondrial mass or expressions of related genes not examined in this study may also contribute to changes in CS activity, COX II level, and CoQ_10_ level. Furthermore, mitochondria–endoplasmic reticulum (ER) contacts are important in regulating mitochondrial functions and in tumorigenesis [40]. CoQ biosynthesis was recently postulated to occur at sites near mitochondria-ER contact sites [41]. The findings regarding the abnormalities of mitochondria-associated membranes in human astrocytomas reported by Arismendi-Morillo et al. [16] may partially explain the mechanism for decreased CoQ_10_ levels in astrocytomas.

We employed a quantitative approach to compare the protein levels of PDSS2 and several COQ proteins among samples across different Western blotting experiments. The inverse correlation between decreased protein levels of COQ3-a, COQ3-b, COQ5, COQ6, COQ7, COQ8A, and COQ9 and malignancy could be related to abnormal mitochondrial status, including decreased formation of the mature forms caused by mitochondrial dysfunction, alterations of mitochondrial mass, and mtDNA-related defects, but we could not exclude the contribution of any alterations in mRNA levels. Moreover, the positive correlation between PDSS2-b or PDSS2-c levels and the malignancy and the strong upregulation of PDSS2-b indicate an unusual signature of this protein in astrocytomas. Because two PDSS proteins are responsible for forming polyprenyl side chains at the first step of the terminal biosynthetic pathway before the reactions catalyzed by other COQ proteins [4], we speculate that PDSS2 upregulation may be a compensatory response for low activities at the later biosynthetic steps caused by a decrease in multiple COQ proteins. The high PDSS2 levels in Grade IV astrocytomas might explain why CoQ_10_ levels in Grade IV astrocytomas slightly rebounded from the decline in Grade III astrocytomas. It is interesting to note that Kuhl et al. [38] also discovered upregulated PDSS2 protein levels and downregulated levels of various COQ proteins in the *Lrpprc* knockout mice with defects in posttranslational regulation of mtDNA and Complex IV deficiency, although the mechanism was also unknown. Moreover, we previously did not evaluate PDSS-c level in the human 143B cells because the abundance was too low to be reliably detected [7]. However, the abundance was easily detectable in the human brain tissues, although PDSS2-c was still less abundant than PDSS2-b. Changes in PDSS2-c among the different groups were not identical to those for PDSS2-b, although they were similar. The correlation of PDSS2-b with higher tumor grades was stronger than that for PDSS2-c. The level of PDSS2-c, but not PDSS2-b, was inversely correlated with CS activity and COX II level. The regulatory mechanism for the PDSS2-b protein level therefore appears to be different from that for the PDSS2-c isoform. On the other hand, Coq4 was proposed to be a central organizer for the putative CoQ synthome and to bind the polyisoprenyl tail of CoQ-intermediates in yeast [4]. It was possible that the tendency of upregulated COQ4 level in Grade III astrocytomas was also a response to downregulated CoQ_10_ levels and that effect was diminished when PDSS2 level was increased or other abnormalities appeared. In addition, Coq8 is required for the phosphorylation of Coq3, Coq5, and Coq7 polypeptides in yeast [4]. The findings of the associations of lower COQ8A:CS or COQ8A:COX II ratio with poor outcomes for Grade IV astrocytomas might be related to the potential regulatory roles of COQ8A.

Our findings regarding PDSS2 were opposite to the findings of decreased PDSS2 protein level reported by other groups for several cancer tissues [22,23,24,25]. However, these studies did not indicate the exact protein forms or sizes of PDSS bands in Western blot results or did not verify whether the antibody used could detect specific protein bands [22,23,25]. In addition, many studies have conducted IHC to detect PDSS2 in cancer tissues [22,23,24,25], meaning that the results have been relatively nonquantitative; the IHC signals could have been from nonspecific proteins. Because PDSS1 and PDSS2 should together form a functional protein, the upregulation of *PDSS1* gene and PDSS1 protein in triple-negative breast cancer tissues reported by Yu et al. [27] might indicate a different role of PDSS proteins, although that study did not measure CoQ_10_ level. The discrepancies between our findings and the results from those reports on PDSS2 may also have been partially caused by the use of different control samples, the use of various grades of cancer tissues in this study, or the studies being on differing cancers.

Taken together, we have demonstrated that lower CoQ_10_ levels and protein levels of COQ3, COQ5, COQ6, COQ7, COQ8A, and COQ9 were associated with higher tumor grades and lower CS activity or COX II level in human astrocytomas. That could be a condition of CoQ_10_ deficiency or dysregulation of COQ proteins secondary to mitochondrial dysfunction. The mechanism of the positive correlation between PDSS2-b or PDSS2-c upregulation and malignancy is currently unknown, but it was not found to be associated with changes in CoQ_10_ level and may be a compensatory alteration in response to decreased levels of most COQ proteins. Moreover, low value of CS- or COX II-normalized COQ8A level might be a novel indicator of poor outcomes for patients with Grade IV astrocytomas. The results also suggest the association of impaired CoQ_10_ maintenance with tumorigenesis or progression of human astrocytomas. Therefore, the roles of endogenous CoQ_10_, PDSS2 proteins, and COQ proteins in astrocytomas or human cancers are worth further investigation. Although the limitation of this study was that we could not perform ultrastructural analysis, biochemical analysis, or molecular analysis concurrently to elucidate the primary mechanism of mitochondrial abnormalities, such as decreased mitochondrial mass, decreased mitochondrial energy production, or mtDNA mutations, that could lead to decreased CoQ_10_ levels or alterations in PDSS2 and COQ protein levels because of limitations in conducting a clinical study, the findings obtained with the use of patient specimens are novel and valuable.

## Figures and Tables

**Table 1:** Summary of patient information and analyses performed.

Group	Patient	Conditions	1-yr GOS	CS *	CoQ_10_ and α-Tocopherol	PDSS2 and COQ Proteins
Nontumor control	1	TBI	NA.	+	+	+
	2	TBI	NA.	+	+	+
	3	TBI	NA.	+	+	+
	4	TBI	NA.	+	+	+
	5	TBI	NA.	+	+	+
Low grade	6	Grade II	G	+	+	+
	7	Grade I	M	+	+	+
	8	Grade I	G	+	+	+
	9	Grade II	G	+	+	+
	10	Grade II	G	+	+	+
	11	Grade II	G	+	+	+
	12	Grade II	M	+	+	+
	13	Grade II	G	+	+	+
	14	Grade II	G	+	+	+
Grade III	15	Grade III	D	ND.	+	ND.
	16	Grade III	G	+	+	ND.
	17	Grade III	G	+	+	+
	18	Grade III	G	+	+	+
	19	Grade III	M	+	+	+
	20	Grade III	G	+	+	+
Grade IV	21	Grade IV	D	+	+	+
	22	Grade IV	D	+	+	+
	23	Grade IV	G	+	+	+
	24	Grade IV	S	+	+	+
	25	Grade IV	G	+	+	+
	26	Grade IV	D	+	+	+
	27	Grade IV	S	+	+	+
	28	Grade IV	M	+	+	+
	29	Grade IV	M	+	+	+
	30	Grade IV	S	+	+	+
	31	Grade IV	D	+	+	+
	32	Grade IV	G	+	+	+
	33	Grade IV	D	+	+	+
	34	Grade IV	S	+	+	+
	35	Grade IV	S	+	+	+
	36	Grade IV	M	+	+	+
	37	Grade IV	D	+	+	+
	38	Grade IV	D	+	+	+
	39	Grade IV	D	+	+	+
	40	Grade IV	D	+	+	+

* Data were shown in the previous study [20]. “+” indicates that the analysis was performed for this sample. Grades I to IV refer to the grading of astrocytoma malignancy. Y and N indicate the presence and absence of the R132H mutation of IDH1 proteins, respectively. G, M, S, V, and D represent good, moderate, severe, vegetative, and dead for the grading of GOS score, respectively. TBI, traumatic brain injury; NA., not applicable; ND., not done; GOS, Glasgow Outcome Scale; CS, citrate synthase; CoQ_10_, coenzyme Q_10_; PDSS, polyprenyl diphosphate synthase subunit.

**Table 2:** Correlations between CS activity or COX II level and the values of different parameters.

Parameters	CS	COX II
CoQ_10_	0.410 **	0.229
α-tocopherol	0.076	0.162
PDSS2-b	−0.148	−0.015
PDSS2-c	−0.469 ***	−0.506 ***
COQ3-a	0.780 ***	0.640 ***
COQ3-b	0.751 ***	0.712 ***
COQ4	0.206	0.530 ***
COQ5	0.563 ***	0.719 ***
COQ6	0.684 ***	0.757 ***
COQ7	0.653 **	0.769 ***
COQ8A	0.449 ***	0.512 ***
COQ9	0.613 ***	0.777 ***
COX II	0.460 ***	NA.

Data in the middle and right columns are the coefficients for the correlation of different parameters with CS activity and COX II level, respectively, for all the samples. NA., not applicable. ** *p* < 0.01; *** *p* < 0.005.

**Table 3:** Correlations between tumor grades or GOS scores and the values of different parameters.

Parameters	Malignancy		1-Year GOS (Grade IV)
	All Groups	No Grade IV	
CoQ_10_	−0.405 **	−0.704 ***	0.218
α-tocopherol	−0.047	−0.408	0.307
PDSS2-b	0.541 ***	0.769 ***	−0.016
PDSS2-c	0.375 *	−0.101	−0.290
COQ3-a	−0.504 ***	−0.624 **	0.277
COQ3-b	−0.530 ***	−0.406	0.029
COQ4	−0.217	0.402	0.052
COQ5	−0.555 ***	−0.163	0.277
COQ6	−0.544 ***	−0.400	0.128
COQ7	−0.434 **	−0.236	−0.360
COQ8A	−0.453 ***	−0.295	0.019
COQ9	−0.402 *	−0.281	0.285
COX II	−0.334 *	−0.186	−0.123

Data in the middle two columns are the coefficients for the correlation between the malignancy grades and values of different parameters for all the samples (all groups) and the samples without Grade IV astrocytomas (no Grade IV), respectively. Data in the right column show the coefficients for the correlation between 1-year GOS scores and values of various parameters only for Grade IV astrocytomas. * *p* < 0.05; ** *p* < 0.01; *** *p* < 0.005.

**Table 4:** Correlations between tumor grades or GOS scores and the values of different parameters normalized by CS activity or COX II level.

Parameters	Malignancy		1-Year GOS (Grade IV)
	All Groups	No Grade IV	
CoQ_10_/CS	0.198	−0.278	−0.123
PDSS2-b/CS	0.664 ***	0.751 ***	−0.024
PDSS2-c/CS	0.547 *	0.336	−0.317
COQ3-a/CS	−0.344 *	−0.452	0.220
COQ3-b/CS	−0.258	−0.092	0.157
COQ4/CS	0.143	0.660 **	−0.092
COQ5/CS	−0.162	0.187	−0.068
COQ6/CS	−0.308	−0.048	0.140
COQ7/CS	−0.088	0.284	−0.038
COQ8A/CS	0.131	0.229	−0.567 **
COQ9/CS	0.033	0.011	−0.162
COX II/CS	−0.022	0.274	0.279
CoQ_10_/COX II	0.068	−0.368	−0.167
PDSS2-b/COX II	0.714 ***	0.645 ***	−0.136
PDSS2-c/COX II	0.440 **	0.123	−0.335
COQ3-a/COX II	−0.359 *	−0.448	0.016
COQ3-b/COX II	−0.428 **	−0.407	0.092
COQ4/COX II	-0.243	−0.657 ***	−0.332
COQ5/COX II	−0.382 *	−0.306	−0.261
COQ6/COX II	−0.371 *	−0.483 *	−0.221
COQ7/COX II	−0.236	−0.333	−0.425
COQ8A/COX II	0.015	−0.209	−0.666 ***
COQ9/COX II	0.043	−0.306	−0.424

Data in the middle columns are the coefficients for the correlation between the malignancy grades and values of different parameters for all the samples (all groups) and the samples without Grade IV astrocytomas (no Grade IV), respectively. Data in the right column show the coefficients for the correlation between 1-year GOS scores and values of various parameters only for Grade IV astrocytomas. * *p* < 0.05; ** *p* < 0.01; *** *p* < 0.005.

## Data Availability

Not applicable.

## Supplementary Materials

The following are available online at https://www.mdpi.com/article/10.3390/biom12020336/s1, Figure S1: Levels of PDSS2, COQ proteins, COX II, and CoQ_10_ normalized by CS activity, Figure S2: Levels of PDSS2, COQ proteins, and CoQ_10_ normalized by COX II level.
