# Supplementary material for: Levels of Coenzyme Q10 and Several COQ Proteins in Human Astrocytoma Tissues Are Inversely Correlated with Malignancy
Source: Biomolecules. 2022 Feb 20;12(2):336. doi: 10.3390/biom12020336 (PMC8869183; doi:10.3390/biom12020336)
Supplement: Supplementary file 1 [file biomolecules-12-00336-s001.zip › biomolecules-1497839-supplementary.pdf]

Supplemental Figures

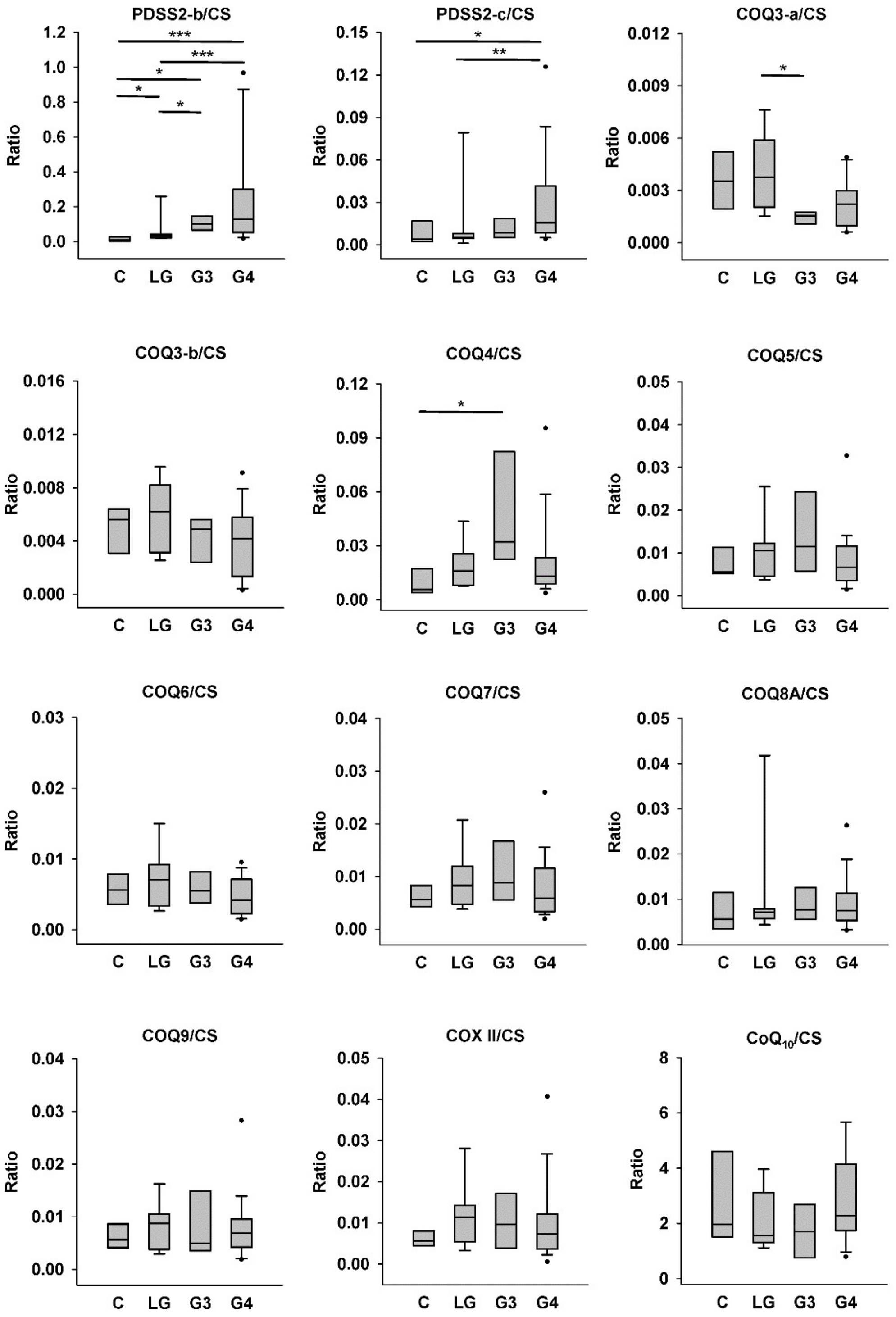

Supplemental Figure S1. Levels of PDSS2, COQ proteins, COX II, and CoQ_{10} normalized by CS activity. C, controls; LG, low-grade astrocytomas; G3, Grade III astrocytomas; G4, Grade IV astrocytomas. * $P < 0.05$; ** $P < 0.01$; *** $P < 0.005$.

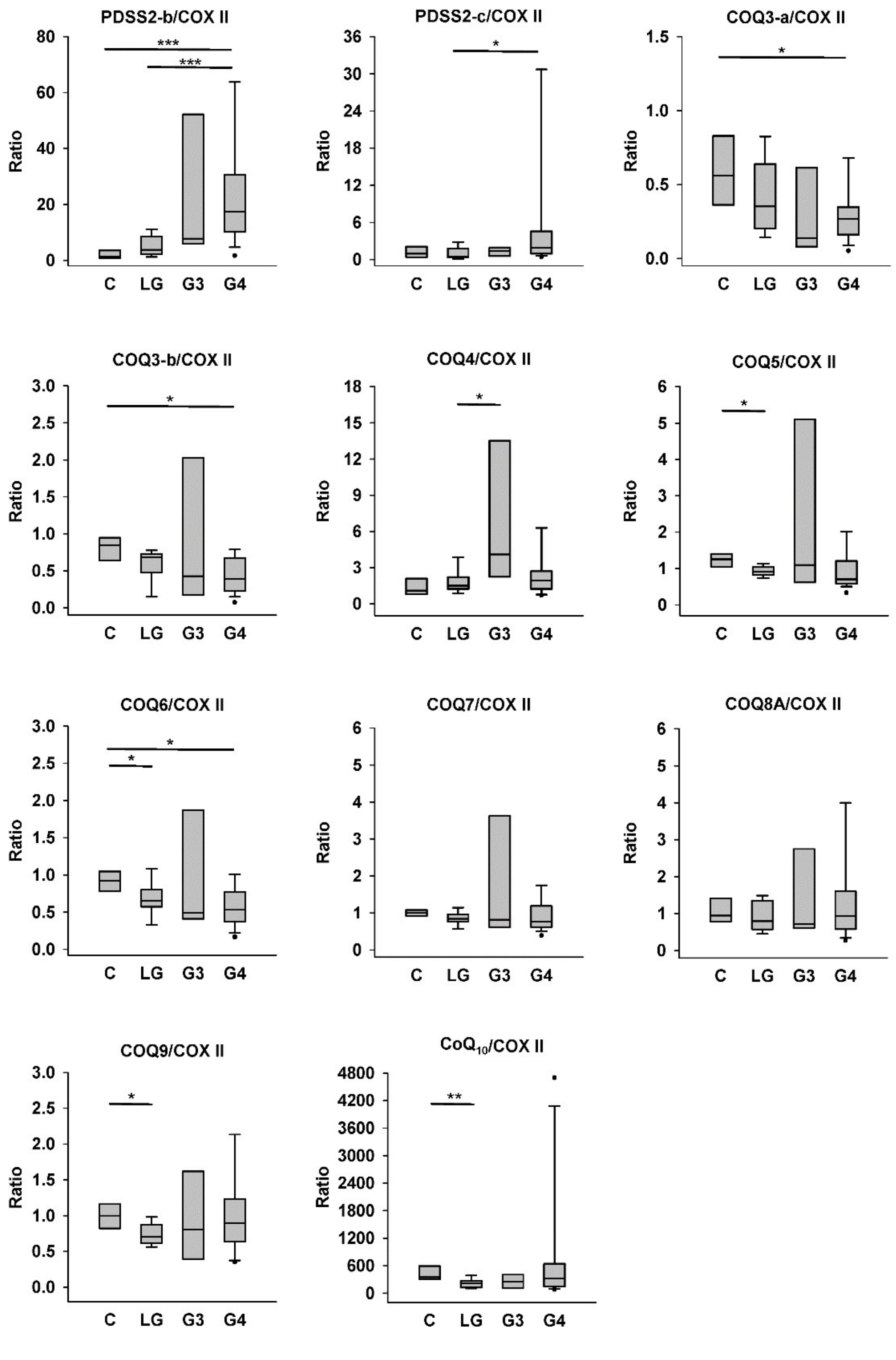

Supplemental Figure S2. Levels of PDSS2, COQ proteins, and CoQ_{10} normalized by COX II level. C, controls; LG, low-grade astrocytomas; G3, Grade III astrocytomas; G4, Grade IV astrocytomas. * $P < 0.05$; *** $P < 0.005$.
